# A novel nonsense variant in *ARID1B* causing simultaneous RNA decay and exon skipping is associated with Coffin-Siris syndrome

**DOI:** 10.1038/s41439-022-00203-y

**Published:** 2022-07-25

**Authors:** Viktoriia Sofronova, Yu Fukushima, Mitsuo Masuno, Mami Naka, Miho Nagata, Yasuki Ishihara, Yohei Miyashita, Yoshihiro Asano, Takahito Moriwaki, Rina Iwata, Seigo Terawaki, Yasuko Yamanouchi, Takanobu Otomo

**Affiliations:** 1 Department of Molecular and Genetic Medicine, Kawasaki Medical School, Kurashiki, Japan; 2 Laboratory of Molecular Medicine and Human Genetics, North-Eastern Federal University, Yakutsk, Russia; 3 Division of Neonatology, National Hospital Organization Okayama Medical Center, Okayama, Japan; 4 Department of Medical Genetics, Kawasaki Medical School Hospital, Kurashiki, Japan; 5 Genetic Counseling Program, Graduate School of Health and Welfare, Kawasaki University of Medical Welfare, Kurashiki, Japan; 6 Department of Cardiovascular Medicine (IRUD Analysis Center), Osaka University Graduate School of Medicine, Suita, Japan

**Keywords:** Genetics research, Sequencing

## Abstract

Coffin-Siris syndrome (CSS) is a congenital disorder that is characterized by an absent/hypoplastic fifth distal phalanx, psychomotor developmental delay, and coarse facial features. One of the causative genes, *ARID1B* (AT-rich interactive domain-containing protein 1B), encodes components of the BAF chromatin remodeling complexes. Here, we report a case of a 3-year 8-month-old male with a novel nonsense variant (NM_001374820.1:c.4282C > T, p.(Gln1428*)) in the *ARID1B* gene, which was identified with whole-exome sequencing. He showed clinical symptoms of cleft soft palate, distinctive facial features (flat nasal bridge, thick eyebrows, and long eyelashes), right cryptorchidism, and hypertrichosis that partially overlapped with CSS. One of the most characteristic features of CSS is absent/hypoplastic fifth distal phalanx. He showed no obvious clinical finding in the lengths of his fingers or in the formation of his fingernails. However, radiographic analyses of the metacarpophalangeal bones revealed shortening of all the distal phalanges and fifth middle phalanges, suggesting brachydactyly. We performed mRNA analyses and revealed that both nonsense-mediated decay and nonsense-associated altered splicing were simultaneously caused by the c.4282C > T nonsense variant. The proband’s clinical manifestations fit the previously reported criteria of disease for CSS or intellectual disability with *ARID1B* variant. Altogether, we suggest that c.4282C > T is a pathogenic variant that causes this clinical phenotype.

## Coffin-Siris syndrome: New mutation implicated

Genomic sequencing has revealed a new causative mutation for Coffin-Siris syndrome (CSS), a very rare disease characterized by developmental delays, intellectual disability and various anatomical abnormalities. A team from Japan led by Takanobu Otomo of Kawasaki Medical School in Kurashiki performed genetic sequencing on a young boy who showed many hallmark features of CSS. The researchers identified a previously undescribed mutation in *ARID1B*, a gene previously linked to the disease. The mutation introduced a premature stop signal into the *ARID1B* gene transcript. The boy’s cells responded to the genetic defect by activating two kinds of RNA surveillance mechanisms: one that helped eliminate the faulty transcript, and another designed to alter splicing patterns. The findings could help the medical community diagnosis other cases of CSS and ultimately find treatments.

## Introduction

*ARID1B* (AT-rich interactive domain-containing protein 1B [MIM *614556]) is recognized as the most common causative gene for Coffin-Siris syndrome (CSS; MIM #135900), which is inherited in an autosomal dominant manner. CSS was first described in 1970 by G.S. Coffin and E. Siris^1^ as a rare congenital disorder with mild-to-moderate intellectual disability (ID), aplasia or hypoplasia of the distal phalanx or nail of the fifth and additional digits, severe speech impairment, coarse facial features, hypertrichosis, and agenesis of the corpus callosum^2,3^. However, previous research and recent advancements in molecular diagnostic techniques have revealed that CSS is a clinically and genetically heterogeneous disorder^4^. CSS is caused not only by variants of the *ARID1B* gene but also by other genes, including *ARID1A*, *ARID2*, *DPF2*, *PHF6*, *SMARCA2*, *SMARCA4*, *SMARCB1*, *SMARCC2*, *SMARCE1*, *SOX4*, and *SOX11*. Thus, genetic testing of these genes is one of the diagnostic criteria for CSS^2,5–7^.

The recent developments from genomic analyses has revealed that *ARID1B* variants are a common cause of ID. They are associated not only with CSS but also with Nicolaides-Baraitser syndrome^5^. Most of these variants are *de novo* variants, and no specific gene hotspots have been suggested to cause haploinsufficiency^4,7,8^. *ARID1B* and the other genes mentioned above encode components of the BAF chromatin remodeling complexes^9^, and their dysfunction is thought to affect the expression of many genes during development.

In this study, we describe a case of CSS caused by a novel nonsense variant in the *ARID1B* gene. The examination of the mRNA revealed that two mechanisms, nonsense-mediated decay (NMD) and nonsense-associated altered splicing (NAS),^10,11^ are simultaneously caused by this nonsense variant.

## Materials and Methods

### Whole-exome sequencing (WES)

In the research project “Initiative on Rare and Undiagnosed Diseases; IRUD,” whole-exome sequencing using a trio sample was performed. Total genomic DNA was extracted using the Genomic DNA extraction kit (Biologica Co.) from the individuals’ peripheral blood. WES analysis was performed using the SureSelect Human All Exon V6 kit and SureSelectXT Low Input (Agilent Technology) for capturing and a HiSeq 3000 (Illumina, San Diego, CA, USA) for sequencing with 100 bp paired-end reads. Sequenced data were mapped to their location in the build of the Human Genome annotation (GRCh37/hg19). Variants, including single-nucleotide variants and small insertions and deletions, were called using the Genome Analysis Toolkit and were annotated using ANNOVAR.

### Establishment of lymphoblastoid cell lines (LCLs), cell culture and cycloheximide (CHX) treatment

LCLs were established from peripheral blood samples using a commercial service provided by the SRL company (Tokyo, Japan). LCLs were cultured with RPMI-1640 medium (Wako) supplemented with 10% fetal bovine serum (Sigma) and penicillin-streptomycin (Nacalai) at 37 °C in a 5% CO_2_ incubator. For the analyses of RNA processing, LCLs were treated with 50 μg/mL CHX (Nacalai) or DMSO (as a vehicle for CHX nontreated cells) for 6 hours before extracting total RNA.

### Preparation of genomic DNA and cDNA

Genomic DNA was extracted from ~1 × 10^5^ LCLs using Quick Extract DNA Extraction Solution (Lucigen) according to the manufacturer’s protocol. Total RNA was extracted from ~3 × 10^6^ LCLs using a FastGene RNA Basic Kit (Nippon Genetics) according to the manufacturer’s standard protocol. RNA quality and quantity were determined by a NanoDrop spectrophotometer (Thermo Fisher Scientific, Waltham, MA, USA). Reverse transcription was performed to prepare cDNA with 0.2 μg of total RNA using a Prime Script 1st strand cDNA Synthesis kit (Takara) according to the manufacturer’s protocol.

### PCR and Sanger sequencing

PCR was performed to generate a fragment spanning exon 17 of *ARID1B* from genomic DNA or cDNA using PrimeSTAR Max DNA polymerase (Takara) and specific primer pairs for genomic DNA (fw 5′-TAAACATTTTCCATTCATAATGA-3′, rev 5′-CTACACATTCTCCTTAAAGCATT-3′) and cDNA (fw 5′-GGTGGAAGAAGCAGTACAATCA-3′, rev 5′-TTGTACATCTCCTGCTGCTG-3′). PCR products were purified using the Wizard SV Gel and PCR Clean-Up System (Promega). Sanger sequencing of PCR products was performed using a specific primer: fw 5′-CTCTCAGAGGGCCTTTGTCG-3′ for the genomic DNA sequence and fw 5′-AATGAGAAAAGTGCCTGGAA-3′ for the cDNA sequence.

### In silico analyses

Pathogenicity prediction was performed using in silico tools: CADD (https://cadd.gs.washington.edu) and MutationTaster (http://www.mutationtaster.org).

## Results

### Clinical presentation

**Fig. 1:**
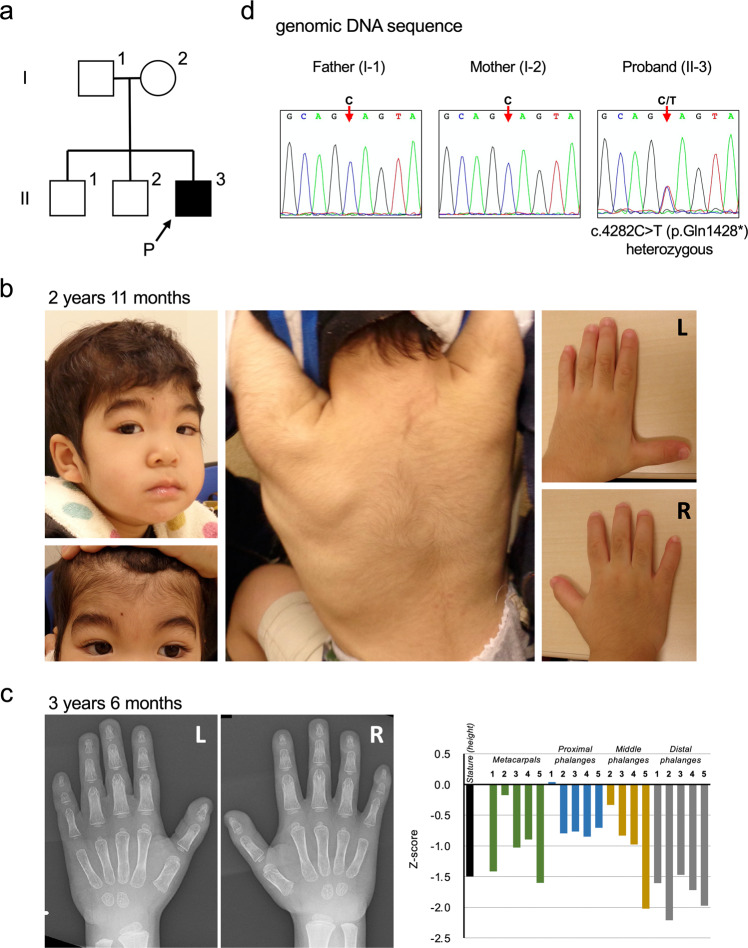
The familial pedigree, clinical features, and genetic analysis. **a** The pedigree of the family. **b** The clinical features of the proband at the age of 2 years 11 months. He showed thick eyebrows, long eyelashes, and hypertrichosis. Morphological abnormalities were not observed in his fingers. Photo usage was approved by the parents of the proband with written informed consent. **c** The radiographic examination of the metacarpophalangeal bones at the age of 3 years 6 months. The lengths of the bones of the left hand were measured, and the Z scores (SD value above or below the expected mean) were calculated according to age-matched Japanese standards^14^. **d** Genetic analysis of the proband and his parents. Sanger sequencing was performed with their genomic DNA. A heterozygous nonsense variant (NM_001374820.1:c.4282C > T, p.(Gln1428*)) was confirmed in the proband.

The proband is a 3-year 8-month-old Japanese male who is the third child of healthy unrelated Japanese parents (Fig. 1a). Neither congenital defects nor psychomotor developmental delay was reported in his two elder brothers and relatives. He was born at 41 weeks and 2 days of gestation, with a weight of 3446 g (+0.2 SD), the height of 52 cm (+1.1 SD), head circumference of 35 cm (+1.0 SD), and Apgar scores of 8/8. At his birth, his father was 46 years old, and his mother was 36 years old. There was no particular problem with the maternal pregnancy and childbirth process. At birth, he had a cleft soft palate, distinctive facial features (flat nasal bridge, thick eyebrows, long eyelashes), right cryptorchidism, and hypertrichosis. Morphological abnormalities in the fingers and fingernails were not reported in his clinical history. MRI was performed for obstructive sleep apnea, and an arachnoid cyst was accidentally found on the dorsal side of the cerebellum. Sleep apnea disappeared immediately, and the size of the arachnoid cyst was reduced by the age of 6 months with follow-up. The karyotype was normal by G-banding. He showed psychomotor developmental delay. He had head control at 6 months, could sit without support at 10 months, could pull up on things at 17 months, could walk without support at 35 months, and does not speak a word at 42 months. The developmental quotient (DQ) was assessed using the Enjoji Scale of Infant Analytical Development at the age of 3 years 8 months; the overall DQ was 26 (physical DQ 30, social DQ 25, and language DQ 22). His clinical manifestations are summarized in Fig. 1b and Table 1 and were compared with the previous reports^2,6,12,13^. At the age of 3 years 6 months, a radiographic examination of his hands was performed (Fig. 1c). The lengths of the metacarpophalangeal bones were measured and evaluated compared with the Japanese standard profiles^14^ to calculate Z scores (SD value above or below the expected mean) (Fig. 1c right). He showed shortening of the middle phalanges (fifth) and distal phalanges (second and fifth) when we took his short stature (−1.5 SD) into account.

**Table 1:** Clinical features of proband in this study and previously reported individuals with ARID1B variants and CSS.

Clinical features		Our case	Gene Reviews^2^	van der Sluijs, et al.^12^		Mannino, et al.^13^	Tsurusaki, et al.^6^
			CSS	*ARID1B*-CSS	*ARID1B*-ID	*ARID1B*-CSS	*ARID1B*-CSS
				(*n* = 79)	(*n* = 64)	(*n* = 49)	(*n* = 20)
*	Developmental delay	+	D	98.6%	100.0%	22%	100%
*	Hypotonia	+	75%	80.3%	82.2%	37%	90%
	Abnormal corpus callosum	−	D	29.0%	28.2%	24%	47%
*	Hirsutism/hypertrichosis	+	95%	94.7%	75.0%	76%	95%
*	Sparse scalp hair	−	60%	62.8%	51.0%	35%	50%
*	Thick eyebrows	+	90%	91.0%	67.9%	N.D.	100%
*	Long eyelashes (prominent)	+	85%	75.9%	44.2%	N.D.	85%
	Coarse appearance	+	95%	90.3%	72.9%	N.D.	100%
*	Flat nasal bridge	+	50%	20.3%	22.0%	N.D.	85%
*	Broad nasal base (wide)	−	50%	43.8%	55.0%	N.D.	90%
	Long philtrum (broad)	−	70%	48.6%	35.1%	N.D.	35%
*	Wide mouth	−	80%	76.0%	58.9%	N.D.	80%
*	Abnormal lips:	−				N.D.	Thick lips 100%
	Upper vermillion, Thick		N.D.	14.7%	30.8%		
	Upper vermillion, Thin		50%	45.3%	21.2%		
	Lower vermillion, Thick		80%	78.9%	55.1%		
	Cleft palate	+	N.D.	5.7%	0.0%	20%	5%
*	Aplasia or hypoplasia of the 5th distal phalanx	−/+**	65–80%	60.6%	9.1%	45%	80%
	Height (< −2 SD)/short stature	−	D	37.1%	21.2%	N.D.	63%
	Feeding problems	+	90%	62.9%	76.3%	12%	70%
	Genital findings (cryptorchidism)	+	D	39.30%	67.60%	N.D.	10%

+ present, − absent, D described without frequency, N.D. not described.

*ARID1B*-CSS: Clinically recognizable Coffin-Siris Syndrome (CSS) cases, which were confirmed by *ARID1B* gene variants.

*ARID1B*-ID: Cases of intellectual disability with *ARID1B* variants, which were identified by large-scale exome sequencing studies. *ARID1B*-ID is a part of *ARID1B*-Related Disorders and patients with *ARID1B*-ID may have no or less physical symptoms of CSS.

*Key features to suspect Coffin-Siris Syndrome^2^.

**Radiographic analyses suggest presence of hypoplasia of phalanges.

### Genetic testing

For the diagnosis, peripheral blood samples from the proband and his parents were subjected to whole-exome sequencing (WES) after informed consent of clinical research. WES identified a variant (NM_001374820.1:c.4282C > T, p.(Gln1428*)) exclusively in the proband that is suspected to be related to CSS. This variant was validated by Sanger sequencing in his family (Fig. 1d). The c.4282C > T variant found in the proband was novel, and its effect has not been reported. In the first report, the IRUD analysis center interpreted this variant as “likely pathogenic” (PS2, PM2, PP3) according to the ACMG guidelines in 2015^15^ using the in silico pathogenicity predictions of CADD (score = 48) and was determined to be “disease causing” by the MutationTaster.

### mRNA analyses

**Fig. 2:**
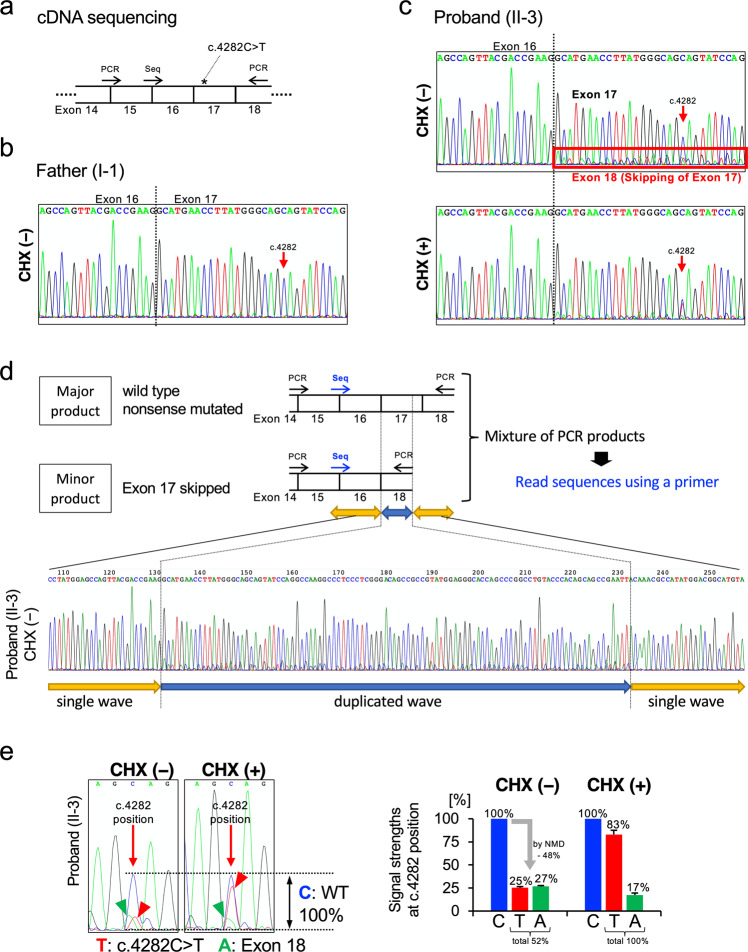
mRNA analyses by Sanger sequencing. **a** Strategy of *ARID1B* cDNA Sanger sequencing. PCR primers (PCR) for amplification and sequencing primer (Seq) are indicated by arrows. **b**–**d** Chromatogram of the *ARID1B* cDNA sequences from the proband (II-3) and his father (I-1). Chromatograms of the forward-direction sequencing from the proband showed a weak signal of the exon 18 sequence following exon 16, which suggests the skipping of exon 17. This exon 18 sequence directly following exon 16 was decreased after treatment with CHX. The c.4282 T signal also increased with CHX treatment. These results suggest that CHX inhibited the NMD and NAS occurring on the mutated allele of the proband. **d** Extended chromatogram of **c**. covering the duplicated range. **e** Quantification of the C/T/A signals at the position of c.4282 from the proband’s cDNA. Using a chromatogram, the heights of the T and A signals from the mutated allele were measured and standardized by the C signal of the wild-type (WT) allele. The mean and SEM from the three independent experiments are shown.

To evaluate the effect of the mutation on pathogenicity, we analyzed the mRNA. Lymphoblastoid cell lines (LCLs) were established from the peripheral blood of the proband (II-3) and his father (I-1). Reverse transcription was performed on RNA extracted from cultured LCLs to prepare cDNA for Sanger sequencing (Fig. 2a). Because cycloheximide (CHX) is reported to inhibit both nonsense-mediated decay (NMD) and nonsense-associated altered splicing (NAS)^16–18^, we treated LCLs with CHX (50 μg/mL) for 6 hours before RNA extraction as a comparison. Without CHX treatment, cDNA from the proband showed the sequences of major C and minor T at the position of c.4282. In addition to these two sequences, another minor wave for the exon 18 sequence was observed at the junction between exon 16 and exon 17 in the forward-direction sequencing (Fig. 2b, c). This suggests the occurrence of exon 17 skipping that is caused by NAS. The overlapping small wave, originating from the cDNA with exon 17 skipping, starts from the exon junction at the terminus of exon 16 but disappears at the end of the PCR fragment (Fig. 2d). This suggests that this small wave is a specific signal from the mRNA with exon 17 skipping. When we treated LCLs from the proband with CHX, the T signal at the c.4282 position increased, and the exon 18 sequence directly following exon 16 decreased (Fig. 2c, e). The *ARID1B* gene contains 20 exons (NM_001374820.1)^6^, and this nonsense variant c.4282C > T is located in exon 17. mRNA containing the premature termination codon (PTC) c.4282C > T becomes the target of NMD. These results suggest that the c.4282C > T nonsense variant causes NMD and NAS simultaneously, and these processes are inhibited by CHX treatment.

## Discussion

Historically, the diagnosis of CSS was completely based on clinical features before the establishment of molecular analyses. Major diagnostic criteria include absent/hypoplastic fifth distal phalanx, psychomotor developmental delay, and coarse facial features^1^. Variants in *ARID1B* are associated with various clinical phenotypes of intellectual disability, which are also recognized as *ARID1B*-related disorders^19^. Morphological abnormalities in the fifth fingers or fingernails, which are the characteristic features of CSS, were not obvious when we performed clinical examination of the surface body in our case. At the age of 2 years 11 months, the length of the patient’s fifth finger was 3.0 cm, and the middle finger was 4.3 cm. Thus, both were similarly (−1 SD) shorter compared with that of the normal values of the Japanese population^20^. Considering the patient’s short stature of 89.5 cm, which is also assessed as −1 SD, it can be concluded that the proband’s fifth fingers are proportional to his height and are not particularly short. However, the radiographic examination of the metacarpophalangeal bones performed at the age of 3 years 6 months suggested hypoplastic distal phalanges and fifth middle phalanges. Recently, a metacarpophalangeal profile in three patients with *ARID1B* variants was reported^21^. In this report, patients showed different patterns of brachydactyly, not only the shortening of the fifth distal phalanges but also the fifth middle phalanges, or a combination of shortened multiple bones. Our patient can be described as a case with mild brachydactyly with a novel *ARID1B* variant. Initially, it was difficult for doctors to diagnose our case as CSS because the complication of fingers was not observed by visual inspection and palpation. A radiographic examination may be useful and considered for the accurate diagnosis of brachydactyly. Both CSS and *ARID1B*‐related disorder have a wide phenotypic spectrum and cannot always be easily separated into two categories. This study suggests that molecular testing, such as WES or gene sequencing panels, are powerful tools for the diagnosis of CSS or *ARID1B*-related disorders.

In this study, we identified a novel nonsense variant c.4282C > T in the *ARID1B* gene and showed the occurrence of both NMD and NAS. The amount of mRNA was quantified based on the Sanger sequencing chromatograms of cDNA (Fig. 2e). mRNA from the proband contained the sequence that originated from the WT allele and two alternative sequences that originated from the mutated allele. We amplified these three products by PCR and read the sequence at the same time using the same primer sets (Supplementary Fig. 1a). We suggest that these two mRNAs are the remnant of NMD and the product of NAS because the amounts of these products were altered by CHX treatment, which inhibits NMD and NAS^16–18^.

Since the heights of the Sanger chromatogram are affected by several factors^22^, we tested a single base primer extension assay using the SNaPshot Primer Extension kit (Thermo Fisher) for the quantification of the RNA. In the SNaPshot method, primers are designed just beside the variant and react with fluorescence-labeled ddNTPs (not dNTPs). The SNaPshot reaction extends one base and stops. By detecting the fluorescence, the extended base is made clear, which is complementary to the variant. When we apply the SNaPshot assay for quantifying our three RNA products, we need to perform two experiments independently and calculate these results to estimate the amount of three RNAs (Supplementary Fig. 1b). This is done because the detection of these three RNA products should be performed at different positions. In our case, the “C” at the position of c.4282 originates from the wild-type allele, and the “T” originates from the nonsense variant of the mutant allele. However, the “A” signal at that position originates not from c.4282 but from the base of the same distance in exon 18. Assay #1 can quantify the amounts of WT and mutated RNA; however, it cannot detect exon 17-skipped RNA. Assay #2 can detect exon 17-skipped RNA; however, it cannot distinguish between WT and mutated RNA. When we compared the chromatograms of genomic DNA obtained from Sanger sequencing and the SNaPshot assay, the SNaPshot chromatogram needed standardization (Supplementary Fig. 1c). We tested the nonsense variant by Assay #1 of SNaPshot, and the results (Supplementary Fig. 1d) were similar to those obtained by Sanger chromatography (Fig. 2e). With these results, we think Sanger sequencing, as a semiquantitative conventional method, is still useful in special situations if we can produce clean data with a deep understanding of the limitations.

The total RNA amount from the mutant allele increased from 52% (25% + 27%) to ~100% (83% + 17%) with CHX treatment (Fig. 2e), suggesting that NMD was completely inhibited by CHX treatment. However, NAS was partly inhibited by CHX treatment because the mRNA with exon 17 skipping remained at ~17%. The recovery of the T signal at the c.4282 position by CHX treatment was caused not only by the inhibition of NMD of nonsense variant-containing mRNA but also by the inhibition of NAS of skipping exon 17 (as a result, the nonsense variant-containing mRNA is produced). It is difficult to discuss the combined effect of the simultaneously occurring NMD and NAS. If a nonsense variant exists in an exon containing the number of bases in multiples of three, NAS may produce a splicing-variant RNA without a frameshift. In those cases, we cannot simply conclude that the effect of the “nonsense variant” is “pathogenic”. This is because the effect of the DNA variant on the phenotype is influenced by the susceptibility of pre-mRNA to NMD and NAS and the stability or functional retention of the exon-skipped short protein. In our case, both transcripts, the “nonsense variant containing” and “exon 17-skipped” mRNAs, had PTC and became targets of NMD. Additionally, even if they are translated, no functional ARID1B proteins are likely produced. This is because both prematurely terminated transcripts lack a nuclear localization signal and BAF250C domain that are important for localizing in the nucleus and forming the BAF complex, respectively. Altogether, we concluded that the nonsense variant c.4282C > T is a pathogenic variant. Our in vitro analyses using LCLs do not necessarily recapitulate the conditions in early development. There is a report that the skipping of exon 17 caused by a synonymous variant is associated with CSS^8^; however, we cannot test how the clinical symptoms of our patient were modified with simultaneous NMD and NAS. These are limitations of this study.

In conclusion, we identified a novel nonsense variant, c.4282C > T, in the *ARID1B* gene. This variant is suggested to be “pathogenic” and to cause the clinical phenotype. This nonsense variant, c.4282C > T, was found to cause NMD and NAS simultaneously.

## Supplementary information

Supplementary Figure 1

## Data Availability

The datasets generated during the current study are available in the LOVD repository, https://databases.lovd.nl/shared/variants/0000831214.
