# Supplementary material for: A novel nonsense variant in ARID1B causing simultaneous RNA decay and exon skipping is associated with Coffin-Siris syndrome
Source: Hum Genome Var. 2022 Jul 25;9:26. doi: 10.1038/s41439-022-00203-y (PMC9314373; doi:10.1038/s41439-022-00203-y)
Supplement: Supplementary file 1 — Supplementary Figure 1 [file 41439_2022_203_MOESM1_ESM.pdf]

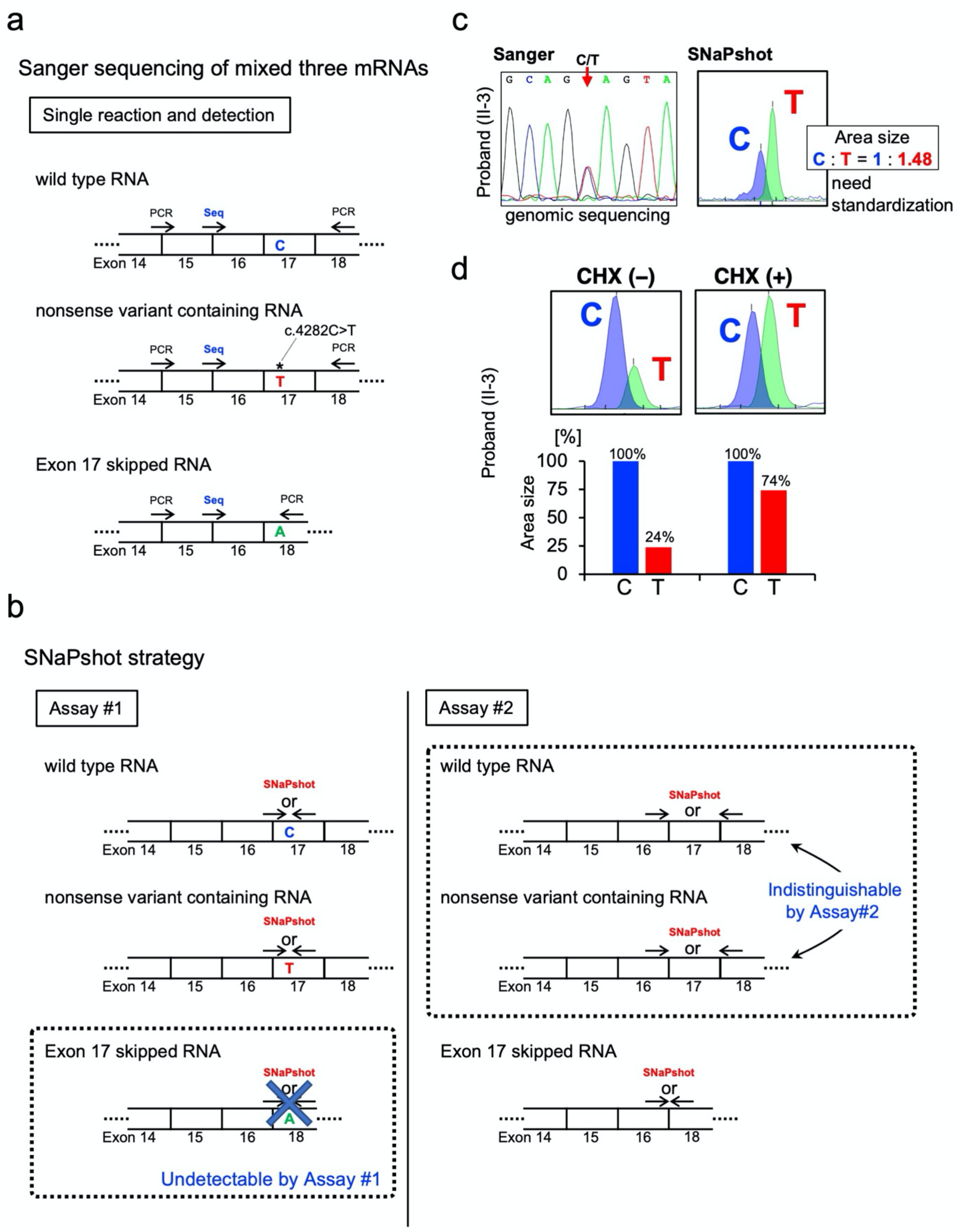

Supplementary Figure 1

Strategies for detecting three different RNA. **a.** The Sanger sequencing method. With this method, three different RNA (cDNA) are amplified by PCR and sequenced at the same time using the same primer set. **b.** The SNaPshot Primer Extension assay. In order to quantify three different RNA (cDNA), two different assays using different primers are performed, and obtained data are summarized to estimate the amounts of three products. **c.** Standardization of SNaPshot data. **d.** Data comparison between Sanger sequencing and SNaPshot assay. These two assays showed similar quantitative results (compare with Fig. 2e).
